# The spatial equity principle in the administrative division of the Central European countries

**DOI:** 10.1371/journal.pone.0187406

**Published:** 2017-11-01

**Authors:** Marián Halás, Pavel Klapka, Vladimír Bačík, Michal Klobučník

**Affiliations:** 1 Department of Geography, Faculty of Science, Palacký University Olomouc, Olomouc, Czech Republic; 2 Department of Human Geography and Demography, Faculty of Natural Sciences, Comenius University in Bratislava, Bratislava, Slovakia; Universidad Nacional de la Plata, ARGENTINA

## Abstract

The paper generally builds on the concept of justice in social science. It attempts to interpret this concept in a geographical and particularly in a spatial context. The paper uses the concept of accessibility to define the principle of *spatial equity*. The main objective of the paper is to propose an approach with which to assess the level of spatial equity in the administrative division of a territory. In order to fulfil this objective the paper theoretically discusses the concept of spatial equity and relates it to other relevant concepts, such as spatial efficiency. The paper proposes some measures of spatial equity and uses the territory of four Central European countries (Austria, the Czech Republic, Hungary, Slovakia) as example of the application of the proposed measures and the corroboration of the proposed approach. The analysis is based on the administrative division of four countries and is carried out at different hierarchical levels as defined by the Nomenclature of Units for Territorial Statistics (NUTS).

## Introduction

The concept of social justice requires fair treatment for all members of society in many areas of life, such as employment, healthcare, education, the right to information, personal freedom, political opinions, leisure time, etc. It is conceived as one of the supreme concepts for a functioning society and is closely connected to the idea of equality [1]. This concept was explicitly introduced to geography by [2], who also developed it further [3]. Since then two views of social justice were established: the distribution of burden and benefits and environmental justice [1]. In its aggregated form social justice is not just concerned with the members of a society in general, but with different social groups based on their economic status, gender and ethnicity. This approach is more interesting for some sub-disciplines of geography as it allows geographers to determine the spatial distribution of social justice (see e.g. [4, 5]) and to apply quantitative techniques in its assessment.

Here the paper gets to its core. As its intent is to look at justice spatially, it builds on the crucial fact that every social group is tied to a space; a social group can be localised and the spatial distribution of social justice can be assessed. This view supports the opinion that social justice and a space are inseparable in certain aspects. Thus there is an opportunity to apply various geographical and quantitative methods, and the paper proceeds from social justice through the relationship between justice and a space to spatial justice, or more precisely to the concept of *spatial equity*. If the concept of justice is handled in spatial terms, the role of distance and accessibility in particular arises immediately (see e.g. [6–10]). Accessibility can be conceived as a wider concept that inherently includes distance. The relationship between accessibility and spatial equity (or social/spatial justice in general) in geographical research is a complex one. It is discussed in connection with the accessibility of different facilities [11–14], mostly related to healthcare (e.g. [15–17]). Besides the location of healthcare and educational facilities, their accessibility to those who need them most is also the issue [18]. Accessibility can therefore be understood as the level of environmental or social exclusion [19, 20].

The paper has three main objectives. Firstly it aims to deal in a strictly “spatial” way with the general issue of justice in social science, using quantitative measures for the assessment. Secondly it aims to come up with and present a general quantitative assessment to the handling of the issue of spatial justice, for the purpose of this paper referred to as spatial equity, in the administrative division of a territory. Thirdly it aims to use, as examples of the application of the proposed approach, the administrative divisions of four Central European countries (Austria, the Czech Republic, Hungary, Slovakia) with different histories and contexts in the development of their administrative divisions. The level of spatial equity is measured and assessed for three different hierarchical levels of the Nomenclature of Units for Territorial Statistics (NUTS). In order to fulfil these objectives the paper builds upon the concept of accessibility, as it is understood in geography, where distance is a crucial feature in a variety of geographical research projects.

The paper maintains that the concept of spatial equity is closely related to the concept of spatial efficiency (see the discussion below). A correct balance between the principles of spatial equity and spatial efficiency is a key issue, particularly for the definition of administrative regions which provide an efficient and equitable administration of a territory, and also fulfil the needs of their citizens. Both principles complement each other, but conceptually can have opposite requirements. Therefore in the theoretical section issues of spatial equity and spatial efficiency are discussed in greater detail, concentrating on their mutual relationship and the factors that help us to assess the level of spatial equity. The next methodological section briefly but sufficiently describes the measures of accessibility that are used to assess the level of spatial equity and the spatial units (administrative units) that are defined for the analysis of the four Central European countries (Austria, the Czech Republic, Hungary, Slovakia). The next section presents the results, which are produced by using the application of theoretical and methodological approaches defined in preceding sections. Finally, the concluding section returns to the objectives of the paper, assesses the validity of the proposed approach and comments on the results from a more general perspective.

## Theoretical background

Spatial equity and spatial efficiency are closely related to the structure of urban and regional systems. The vertical structure of an urban system (a hierarchy) and the horizontal structure of an urban system (spatial distribution) play significant roles. In a pioneering study [21], it is acknowledged that the research into these issues is one of the basic themes of regional science. The close integration of cities in a system means that a significant change in one city can have consequences for the whole system. [21] also points out that the development of computer technology during the quantitative revolution in geography has provided us with a suitable platform for the empirical testing of older neo-classical theories and models and their potential generalisations (e.g. the central place theory). In connection with the structure of urban systems, it is necessary to point out that their current form can be conditioned partly by design and planning, and partly by self-organisation [22].

The terms spatial equity and spatial efficiency have several meanings in the scientific literature. Often they are related to the development of transport networks and transport infrastructure. This is a reaction to the requirements contained in the documents of the European transport and regional policy [23, 24]. In this case the quantitative analysis attempts to assess accessibility and to make the existing transport networks more efficient (see e.g. [25–27]) point out in this respect that the principles and interests of spatial efficiency can be in contrast to the principles and interests of spatial equity. Spatial efficiency concentrates on the good, fast and efficient connection of central urban regions with the highest population densities, while spatial equity, in order to prevent people from being marginalised and spatially isolated, stresses the fact that the most peripheral areas have a minimal level of connection to transport networks.

It is obvious that for various reasons a trade-off between the principles of spatial equity and efficiency is necessary. One reason is the localisation of services, no matter whether they are public or private, although the public ones are more important from the point of view of this paper. Older neoclassical and related neoliberal theories claimed that in the case of commercial services this problem would be handled by the market, laissez-faire [28]. Their opinions were grounded in the presupposition of spatial homogeneity and perfect information flow. Based on these presuppositions many neoclassical models (e.g. Reilly’s law of retail gravitation) were formulated, and they have been usable up to the present day, but their usefulness is limited [29–31].

All these models presume the automatic market fulfilment of the principle of spatial efficiency, but they neglect the principle of spatial equity. Unlike commercial services the principle of spatial equity is or should be absolutely crucial for the public service sector in its widest meaning. Typical examples are the security of basic networks and access to health (accessibility of general practitioners and hospitals for the most peripheral areas) and education (nursery, primary and secondary schools). In the context of massive suburbanisation, access to lower educational institutions (requiring a distance that can be easily managed by children) is an issue, even at the micro regional level, because in suburbanised areas the establishment of public facilities lags behind the residential suburbanisation [32, 33].

In regional policy the setting of an optimal relationship between strategic and insurance regional policy can represent a certain analogy to the trade-off between spatial efficiency and spatial equity. Strategic regional policy primarily supports central areas whose development also stimulates the development of peripheral areas. Insurance regional policy primarily supports regions that are lagging behind [34, 35]. In this context [33] used the terms spatial efficiency and spatial equity when assessing the redistribution of investments between Chinese provinces. However, in this case, both terms, unlike other mentioned meanings, are not related to the accessibility of distant and peripheral areas and regions from regional centres.

In localisation theories the principles of minisum and minimax localisation resemble the principles of spatial efficiency and spatial equity [36, 37]. From the point of view of optimality there are two dominant criteria which determine further approaches: minisum and minimax. To put it simply, the localisation of a new point can be considered as optimal, according to minisum (median), when the sum of the distance from existing points to a new point is minimised. The localisation of a new point can be considered as optimal, according to minimax (centre), when the greatest distance between existing points and the new point is minimised. However, these two criteria may not be sufficient enough. If industrial localisation occurs or any object is added to an environment despite the fact that it should not be localised in the vicinity of human settlements, a rather different objective function is needed. The identification of optimal location is based on the maximisation, not the minimisation of the objective function.

In a similar way to localisation theories, spatial efficiency and spatial equity can also be defined for the assessment of administrative regions. The centre of an administrative region can be considered as the analogy to “a new point” and municipalities in a region as analogies to “existing points”. Spatial efficiency and spatial equity can be quantified by minisum and minimax, but these numbers would result in an undue simplification. [38] maintains that in general spatial efficiency expresses the relationship between spatial location, spatial organisation and economic efficiency. In the context of this paper it is expressed by the requirement to identify such an arrangement, which secures a maximisation, or at least improvement, in benefits for the population of a given territory, i.e. which brings the maximum benefit (level of accessibility) for most of the population of a given territory.

Such an arrangement does not necessarily have to be suitable for all inhabitants of a given territory [39]. The result can be quantifiable but need not be; it would be the minimisation of values of the accessibility measure expressing the total or average accessibility of a centre of administrative region for the population of all of its municipalities. Generally speaking the objective is to search for an administrative arrangement which respects best the natural organisation of a space in relation to the distribution of transport communications, commercial service network, educational, healthcare and social facilities, etc. Quantification also requires the use of alternative accessibility measures to centres (e.g. time accessibility), which can differ from simple metric distances.

The principle of spatial equity can be seen as the application of the principle of social justice on territorial units [38]. For the purpose of this paper the principle expresses the efforts to identify such spatial arrangements which would enable the relative demands of the population of each municipality to be met regardless of their location. If the accessibility of centres of administrative units is analysed, two requirements are reasonable: relatively sufficient access to a centre for the population of all municipalities which form an administrative unit, and sufficient access to the centre of each administrative unit regardless of its location within the unit. In order to fulfil these requirements it is necessary to secure the best possible access for the population of the most distant municipalities in each administrative unit and also to level out regional disparities in the accessibility of centres of administrative units [39, 40].

Spatial efficiency and spatial equity have been taken as two separate issues for a long time. [41] was among the first to put them into context and to look for connections between them. Both principles can be regarded as closely interlinked; in most cases they are able to complement each other, but sometimes they are found to be in conflict. The fact that the largest regional centres in a country have naturally larger hinterlands and tributary regions is a good example. The size differences between the hinterlands of large and small centres can be widely significant. The principle of spatial efficiency tends to respect these differences (e.g. administrative regions at the lowest regional level should reflect the natural distribution of daily population flows the most), but the principle of spatial equity requires that regions at the same hierarchical level be comparable in size (In this respect it should be noted that size refers to an area rather than a population of a given region. Comparable population size cannot be reached, because the population is distributed in an uneven manner and has a relatively high degree of concentration.). Therefore the administrative arrangement should look for a suitable compromise and harmony between the principles of spatial efficiency and spatial equity.

In general spatial equity in the definition of administrative regions depends on the following factors:

1. size of a region,
2. shape (compactness) of a region,
3. location of a centre within a region.

A basic practical rule for the assessment of the principle of spatial equity is based on the requirement that the most distant municipalities in each administrative unit have a comparable distance to the respective centres of those administrative units. The importance of the comparable size of administrative units is obvious. Increasing the size of a unit causes a directly proportional increase in the distance of the most peripheral municipality from the centre of this unit.

**Fig 1:**
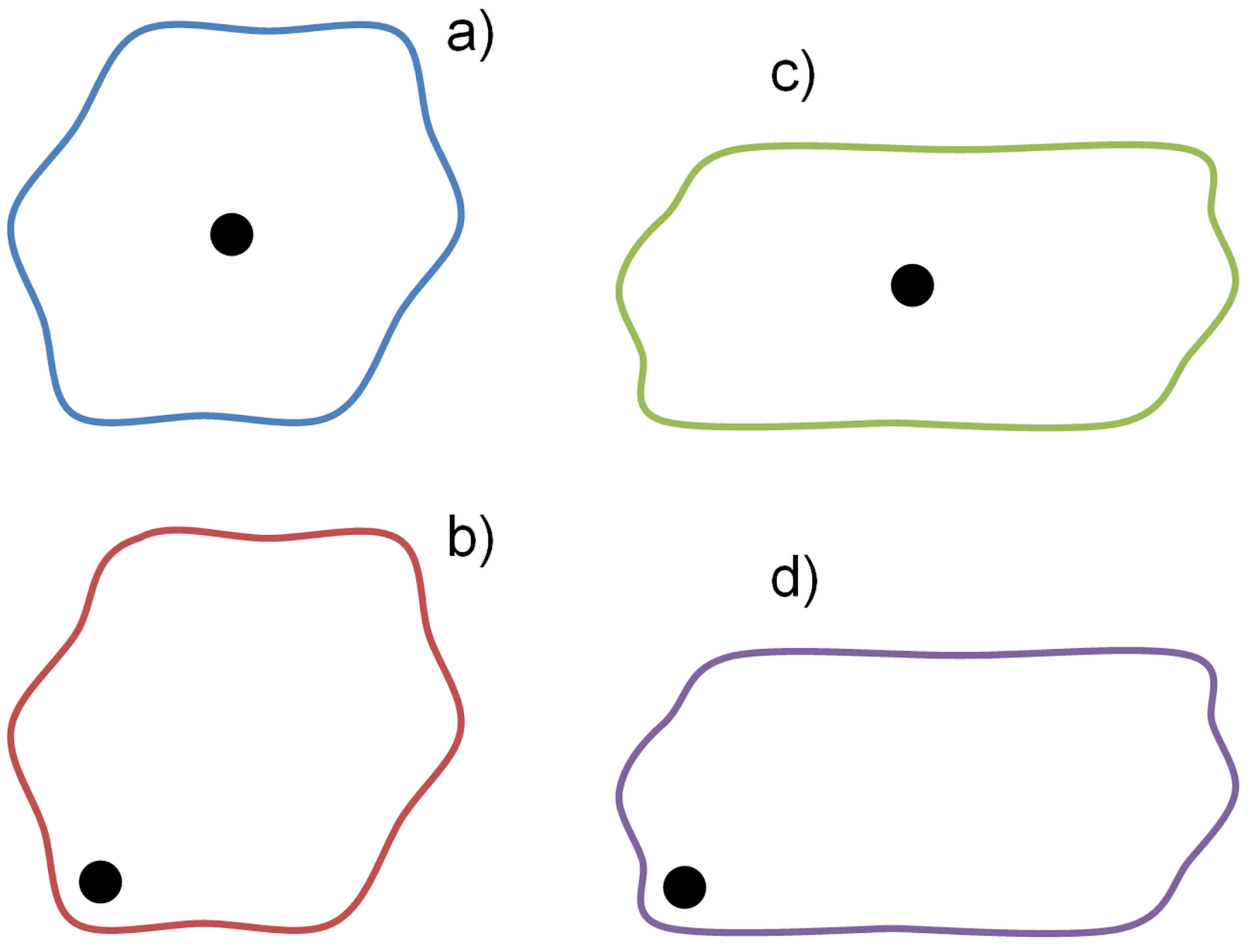
Alternatives for the shape of a unit and for the location of the centre of a unit.

**Fig 2:**
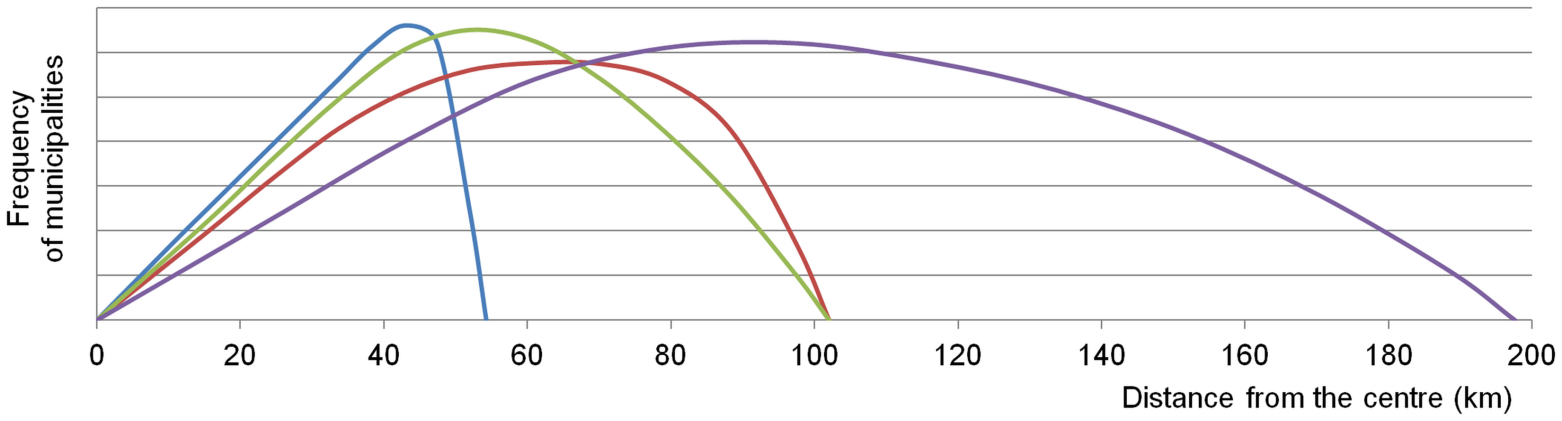
Distribution of municipalities according to the distance from the centre (colours correspond to colours in Fig 1).

The influence of the shape (compactness) of an administrative unit and of the location of its centre on the principle of spatial equity (and also on the principle of spatial efficiency) is given in Fig 1. All four variants depict units with the same size and the same combinations of criteria of compactness of a unit and location of a centre within a unit. The compactness can be high (a, b) or low (c, d), the location of a centre can be central (a, c) or peripheral, eccentric (b, d). Fig 2 gives ideal curves for frequencies of municipalities according to distance from the centre. The area below each curve generally and approximately agrees with the sum of all distances from each municipality to the centre and the differences between all four variants are quite clear.

The frequency of municipalities according to the distance from the centre increases uniformly and then sharply decreases in compact units. The decrease is not as sharp in less compact units; there is a certain number of municipalities more distant from the centre and their location is disadvantaged. Such units often have an elongated or irregular shape. The frequency of municipalities according to the distance from the centre does not increase in every direction in a geographic space, but only along longer axes and to greater distances (Figs 1 and 2).

The location of a centre within a unit is also important in order to assess the spatial equity and spatial efficiency. A relatively central location for the centre is an advantage in both respects; in contrast an eccentric location is a disadvantage. Distances from the most peripheral municipalities to the centre increase and these municipalities are disadvantaged (Figs 1 and 2). When defining administrative units, this can be compensated for by the construction of smaller units, if their centre is located eccentrically.

**Fig 3:**
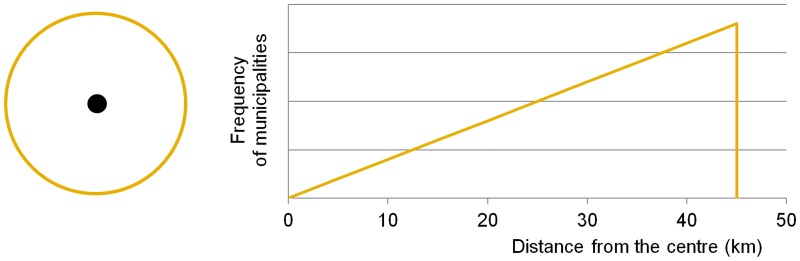
Distribution of municipalities according to the distance from the centre in an ideally compact shape.

A theoretical example may also be given here. As the circle is the most compact shape (A circle is the most compact two-dimensional shape, because of all two-dimensional shapes with the same area it has the smallest circumference, and of all two-dimensional shapes with the same circumference it has the largest area.) and if the centre is located centrally in such a unit, the number of municipalities (in this case taken as points rather than polygons) increases linearly with an increasing distance from the centre (Fig 3). However, geographical units of such a shape hardly exist in practice, because geographical space is naturally heterogeneous and because circular objects cannot be put together to form a plane. This theoretical and ideal shape helps us only as a reference shape, to assess the shape of a unit and the distribution of settlements within this unit, and for further assessment of the spatial equity and spatial efficiency of these units.

The above-mentioned facts suggest that the skewness of the statistical distribution of distances from a reference point is important and that the principle of spatial equity favours negative (left-skewed) asymmetric frequency distribution (Figs 2 and 3). All the above-mentioned examples are theoretical: they take into account relatively regular convex shapes and the regularly dispersed distribution of municipalities. The geographical space is characterised by distinct irregularities: in the distribution of municipalities, in the existence of concave shapes, etc. These irregularities can strengthen the position of municipalities with good access and weaken the position of those with poor access. The difference between both groups increases and therefore the level of spatial equity decreases as the result of irregular spatial distribution of municipalities (settlements).

## Method

The assessment of the accessibility of centres of administrative units in general and also with regard to the principle of spatial equity can involve a wide spectrum of measures [9, 42, 43]. Although there is extensive literature which uses the theory of time geography (e.g. [9, 44, 45]), the paper will employ classical measures of accessibility. The basic parameter is the maximum distance *max(d*_*ci*_*)* between the centre of an administrative unit *c* and the most distant municipality *i* in the administrative unit. From the point of view of spatial equity the variability of this measure is important, with lower variability indicating an increased level of spatial equity and *vice versa*. The integrated distance of the centre of an administrative unit *c* is the weighted sum of the distances of all municipalities from the centre and indicates the spatial efficiency of the administrative arrangement. It is calculated by:

$$ds=\sum_{i=1}^{n}P_{i}\times d_{ci},$$

where *P*_*i*_ is the population of the municipality *i*; *n* is the number of municipalities in the administrative region. Giving weight of distance *d*_*ci*_ more significance in this notation will increase the importance of the access of the most distant municipalities to the centre and take greater account of the requirements of the principle of spatial equity. In this respect it is possible to use the analogy of the moment of inertia to accessibility. This term comes from precise mechanics and in this case it can serve as the measure of population distribution around the centre of an administrative unit [46]. It is calculated by:

$$dm=\sum_{i=1}^{n}P_{i}\times d_{ci}^{2},$$

where the role of distance *d*_*ij*_ is emphasized by the square.

The above-mentioned indices *ds* and *dm* can also be used in a relativised form as the weighted mean distance from the centre:

$$dsr=\left(\sum_{i=1}^{n}P_{i}\times d_{ci}^{}\right)/\sum_{i=1}^{n}P_{i},$$

and the relativised analogy to the moment of inertia:

$$dmr=\left(\sum_{i=1}^{n}P_{i}\times d_{ci}^{2}\right)/\sum_{i=1}^{n}P_{i}.$$

The advantage of relativised indices *dsr* and *dmr* is that their calculation does not disadvantage populationally large regions with high population density (administrative regions tend to have comparable areas, but different populations^1^).

In all preceding examples it is possible to substitute parameters of distance with parameters of accessibility. The simplest way is to substitute metric distance with time distance, which approximates better the actual distribution of municipalities (settlements). It can be supposed that time units better express the quality of a space in terms of its physical geographical characteristics, the quality of transport networks, etc. A greater difference between values of metric distance and time accessibility indicates spatial deformations in the sense that the values of accessibility do not always conform to the values of distance. The measure of spatial deformation can be denoted as relative accessibility and it can be expressed as the proportion of time accessibility *dt*_*ij*_ and metric distance *d*_*ij*_, i.e. *dr* = *dt*_*ij*_/*d*_*ij*_.

The paper will use the above mentioned measures together with the analysis of the municipality-to-centre distance frequencies in order to assess the administrative division, paying particular attention to the spatial equity in four assess the administrative division, paying particular attention to the spatial equity in four countries: Austria, the Czech Republic, Hungary and Slovakia. The analysis will be carried out for three hierarchical levels: LAU 1, NUTS 3 and state level, while the mean level, i.e. the medium level with respect to the hierarchy of regions, which in fact reflects the size of the regions, (NUTS 3) serves also as the regional self-government. Austria is the exception which needs to be explained. NUTS 3 regions in Austria are considerably smaller in size in comparison with the other three countries; in contrast NUTS 2 regions are larger. In this case the NUTS 2 level was favoured because it represents regional self-government in Austria (Bundesländer).

**Fig 4:**
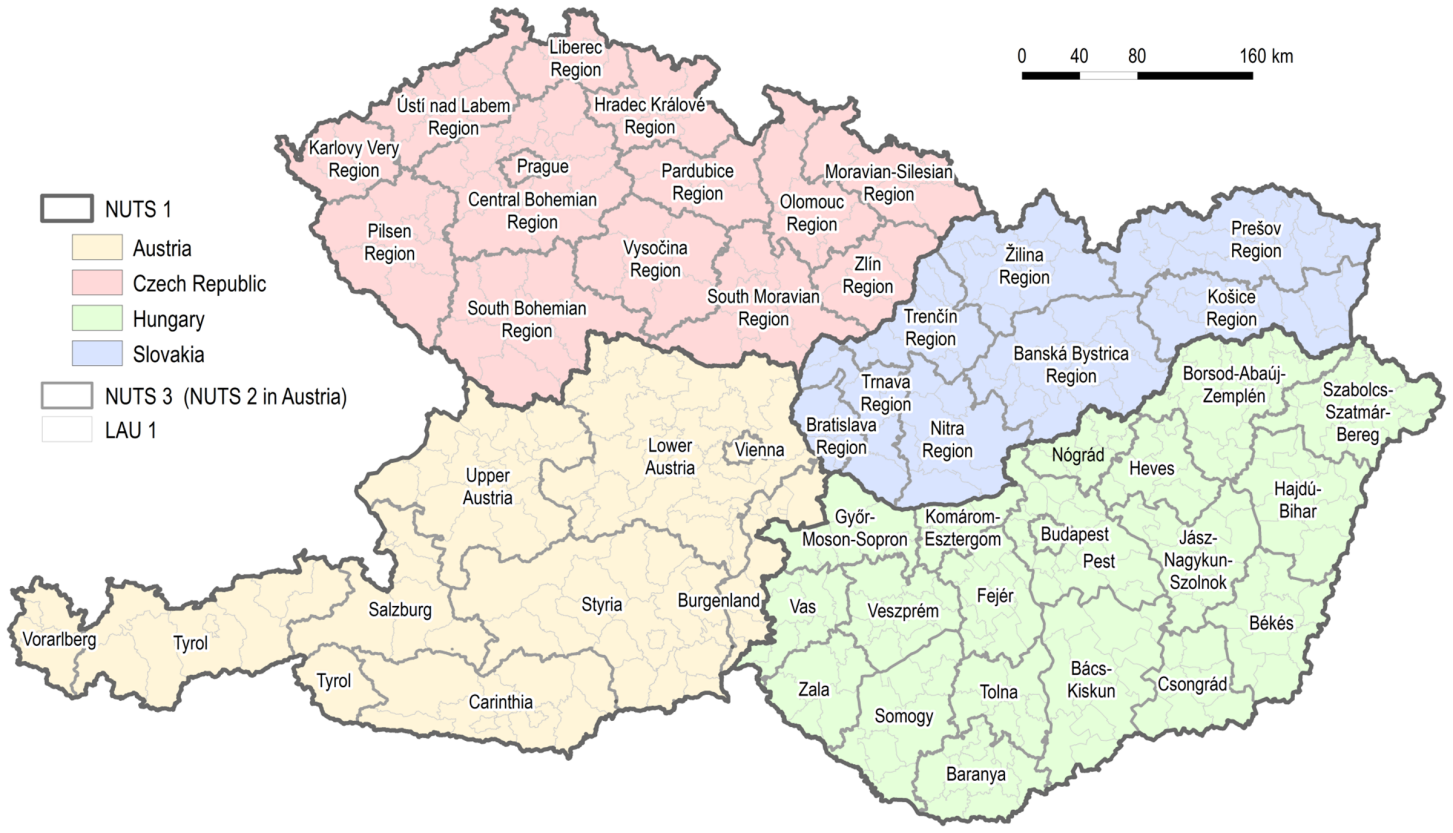
Administrative regions of Austria, the Czech Republic, Hungary and Slovakia.

The terminology of the paper includes two words: municipality and region. As the smallest self-governing spatial units, municipalities vary considerably in their character and area in different countries. For instance in Europe, they are largest in area in the United Kingdom, Ireland and the Scandinavian countries [47]. In Austria, the Czech Republic, Hungary and Slovakia they are comparable in area and are among the smallest units in Europe. Therefore in the current paper they act as basic spatial units for the measurement of distances from centres of administrative regions. In this study regions are primarily understood to be areas with regional self-government (NUTS 3 regions in the Czech Republic, Hungary and Slovakia; NUTS 2 regions in Austria). If the term region is used for areas of different sizes (e.g. LAU 1) and functions (e.g. historical regions), it is properly noted in the text. Fig 4 shows an outline map of Austrian, Czech, Hungarian and Slovak regions. The following maps do not include the names of regions for the sake of clarity.

The distance and accessibility of municipalities to relevant centres (depending on the hierarchical level) in all four countries were acquired by the script which used Google Distance and Google Geocoding services. The localisation of municipalities based on geocoded coordinates was used to gain direct air line distance (Spider Graph tool in MapInfo software). Road distance and time accessibility were obtained in graphical interface using Google Distance API (SQL request). All distances have been measured from the centres of municipalities. A centre is understood to be a centroid of the built-up area of the largest settlement, based on population, within a municipality. In our four countries each municipality has either only one settlement or one settlement is significantly dominant in most other cases.

To conclude the methodological section, the critical methodological issue of the modifiable areal unit problem (MAUP—as addressed for example by [48, 49]) has to be briefly dealt with in the context of the analyses presented. MAUP is an inseparable part of almost any spatial analysis, as in these cases, where arbitrary and modifiable objects (spatial units) are grouped into larger areas. One of the basic questions concerns which form of aggregation should be used to ensure that the results best explain the real spatial distribution of the phenomenon under study. In our case this is important because: the analyses are carried out at several hierarchical levels (a), and the analyses are carried out in four countries (b). With regard to (a), three hierarchical levels have been analysed and, naturally, the administrative regions were the first choice (spatial equity was assessed from the point of view of administrative division). With regard to (b), the use of hierarchical levels in four countries is described and explained in the preceding text.

## Results

Two notes should precede the presentation of the results. Firstly, it must be admitted that the results are not completely comparable for the four analysed countries, however the variability of indices within the countries is very mutually comparable. Even though the whole territory was part of the Habsburg monarchy for hundreds of years, each country currently has its own administrative division that differs in its origin, purpose and parameters. In contrast the EU membership of all the countries gives some basic limits to their administrative division framed by the nomenclature of units for territorial statistics. Basic statistics are given in Table 1. This is a more general note. Secondly the methods described in the preceding section have produced a greater number of results and only some of them are presented in this section. Maps are based on time distance, while graphs are based on metric road distance. The reason for this is that time distance represents a form of spatial deformation, if metric units are taken as the measure that does not deform a space, and maps are better means for the expression of spatial deformation. Metric distance as shown in graphs represents the expression of non-deformed and one of the basic characteristics of a space. Distance decay functions depicted by graphs are most often expressed by a decrease in the volume of interaction depending on the increasing metric distance from a centre. If time distance was shown in graphs, the spatial deformations would remain concealed in comparison to maps. Finally, this intention enables us to present a greater variety of results. In fact the distribution of data on metric distance and time distance (accessibility) is very similar; the correlation coefficient for various hierarchical levels in all four countries reaches values between 0.95 and 0.99. A summary of figures for all countries and for relevant characteristics related to the concept of spatial equity are presented in Table 2.

**Table 1:** Basic population characteristics of administrative units.

Territory	Population (thousands)		
	Total	Average for LAU 1	Average for NUTS 3
Austria	8 430.6	85.2	* 936.7
Czech Republic	10 486.7	136.2	749.1
Hungary	9 985.7	56.7	499.3
Slovakia	5 404.3	75.1	675.5

*NUTS 2.

Source: own calculation.

**Table 2:** Basic characteristics for the spatial equity concept.

Territory	Coefficient of variation (%; LAU 1 level)				Coefficient of variation (%; NUTS 3 level)			
	Maximum distance	Mean distance	Weighted mean distance (*dsr*)	Relativised analogy to moment of inertia (*dmr*)	Maximum distance	Mean distance	Weighted mean distance (*dsr*)	Relativised analogy to moment of inertia (*dmr*)
Austria	56.3	52.7	54.2	74.3	* 40.3	* 40.5	* 45.8	* 55.1
Czech Republic	29.4	26.6	40.4	56.8	34.1	31.9	34.3	42.0
Hungary	32.6	29.9	42.0	64.3	32.1	30.4	34.5	53.3
Slovakia	39.7	36.1	51.0	100.8	23.9	25.9	34.5	50.8

*NUTS 2.

Source: own calculation.

Table 2 shows some basic results for the assessment of spatial equity in the columns for weighted square average distance. At the LAU 1 level the influence of terrain is clearly visible for Austria and Slovakia. Apart from physical geographical conditions, political influences are responsible for the highest value in the case of Slovakia. At the NUTS 3 level the differences between the values are considerably smaller. The territory of the Czech Republic is the most level in terms of the spatial equity at this level.

**Fig 5:**
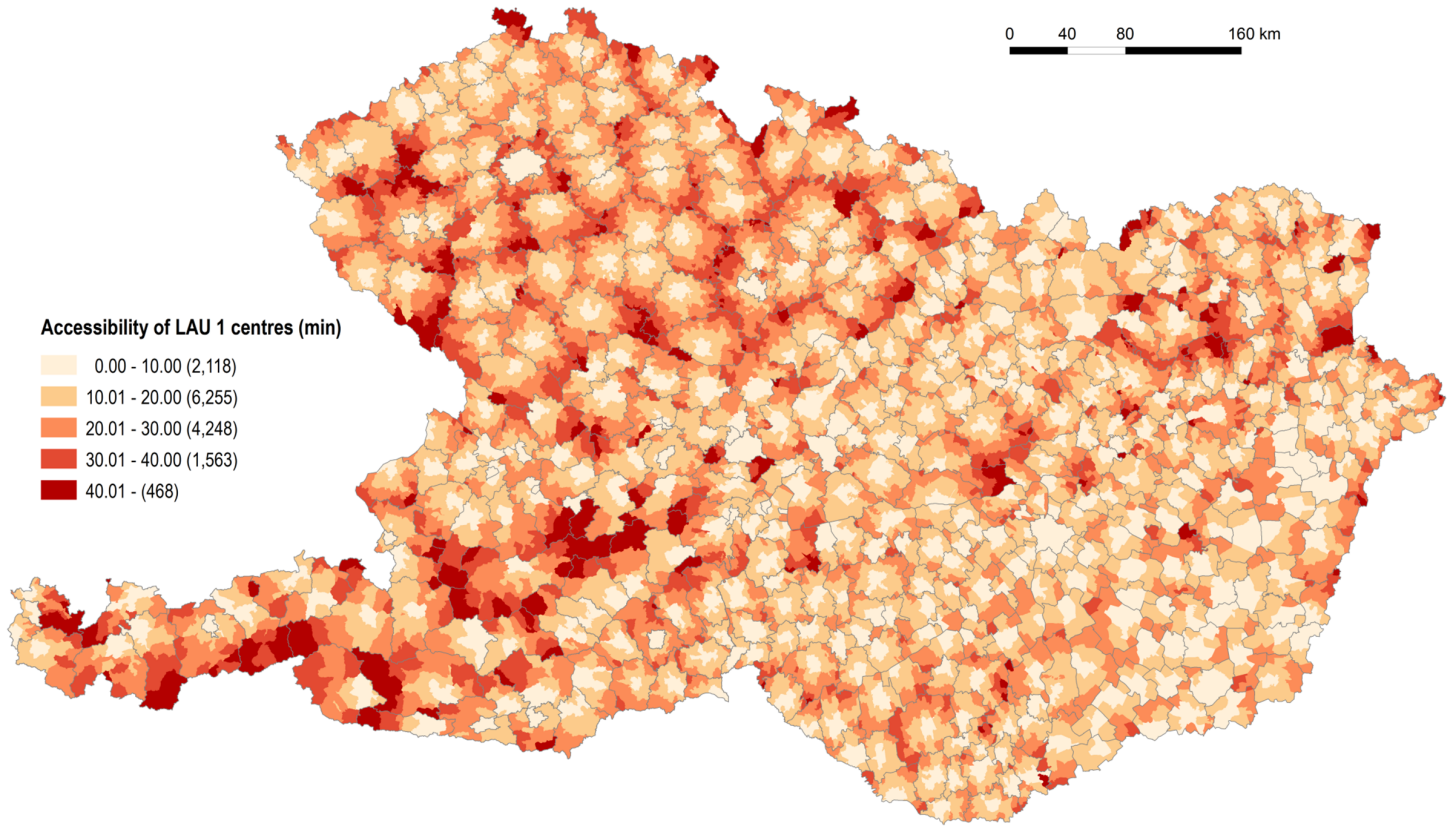
Accessibility of LAU 1 centres by individual road transport. Source: own design.

**Fig 6:**
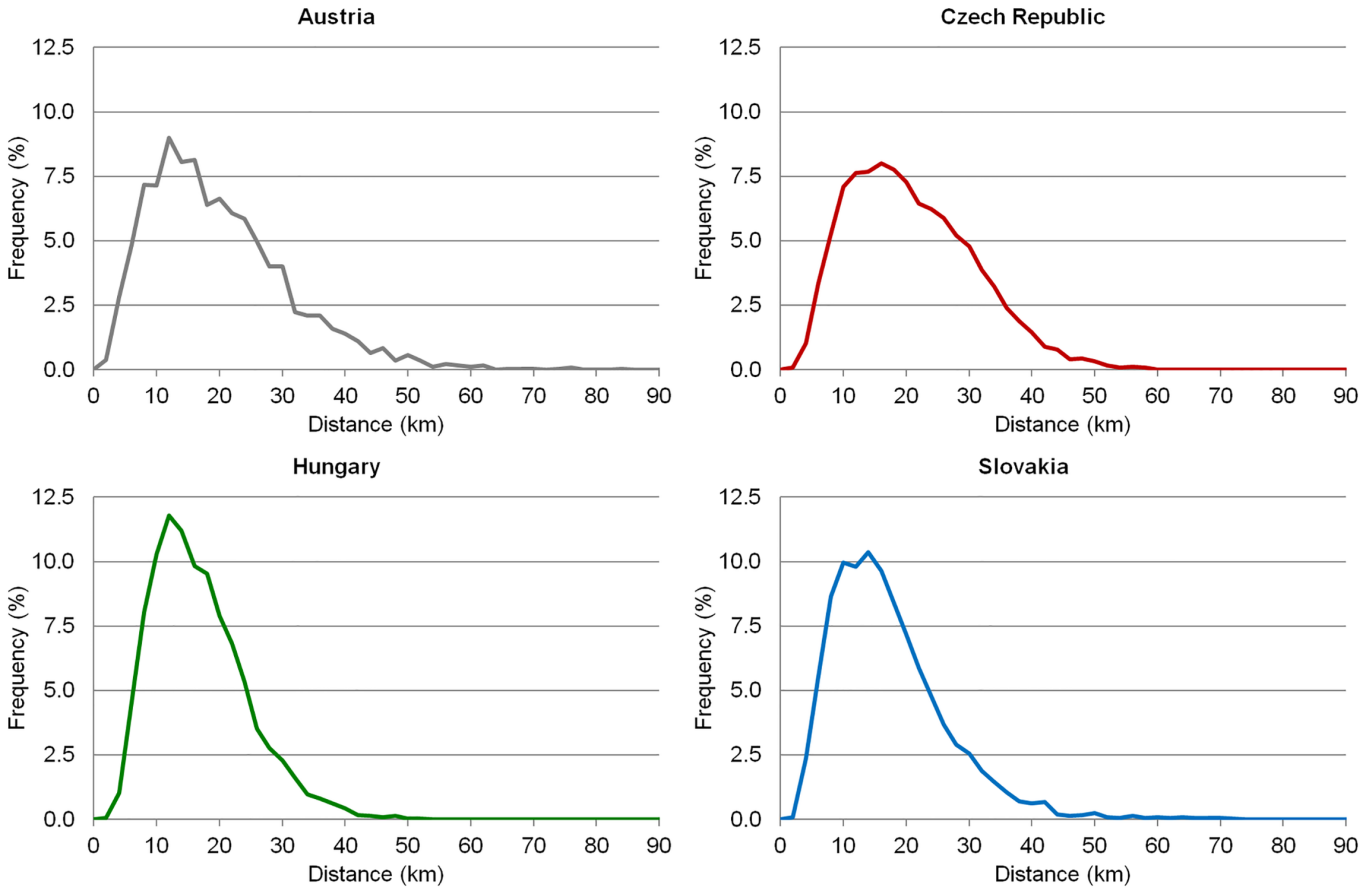
Distribution of municipalities according to distance from LAU 1 centres. Source: own design.

Figs 5 and 6 show the time and metric accessibility from relevant municipalities to the centres of LAU 1 regions. At this hierarchical level the differences between the four analysed countries are not significant (see particularly Fig 6). Three crucial characteristics for accessibility (size and shape of administrative units, location of their centres) do not greatly affect this accessibility. Nevertheless, in Austria the role of physical geographical conditions (terrain in particular) can be seen, similar to the Czech Republic, to effect of the size of LAU 1 regions (they are larger than in the other 2 countries).

**Fig 7:**
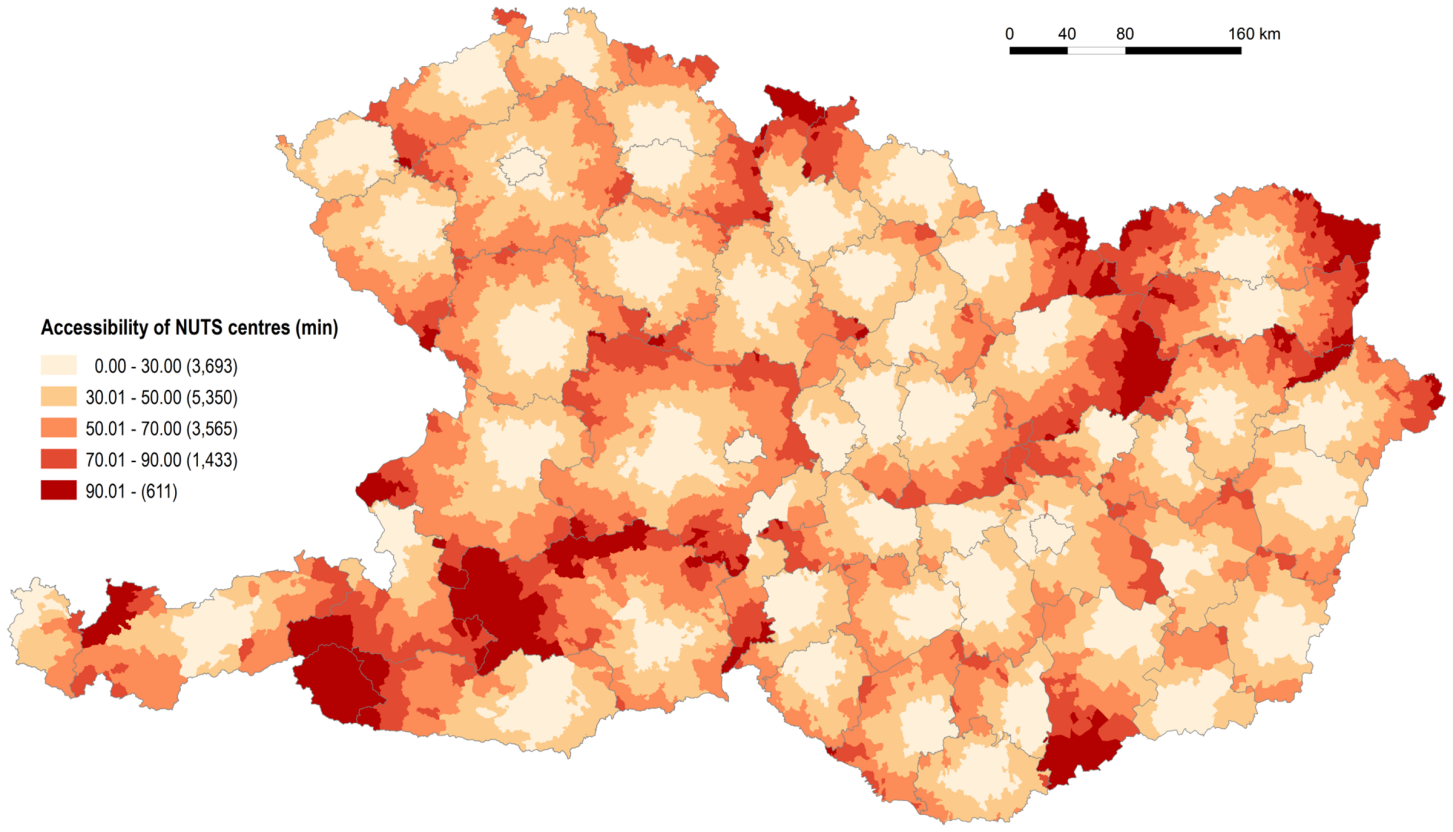
Accessibility of NUTS 3 (NUTS 2 in Austria) centres by individual road transport. Source: own design.

**Fig 8:**
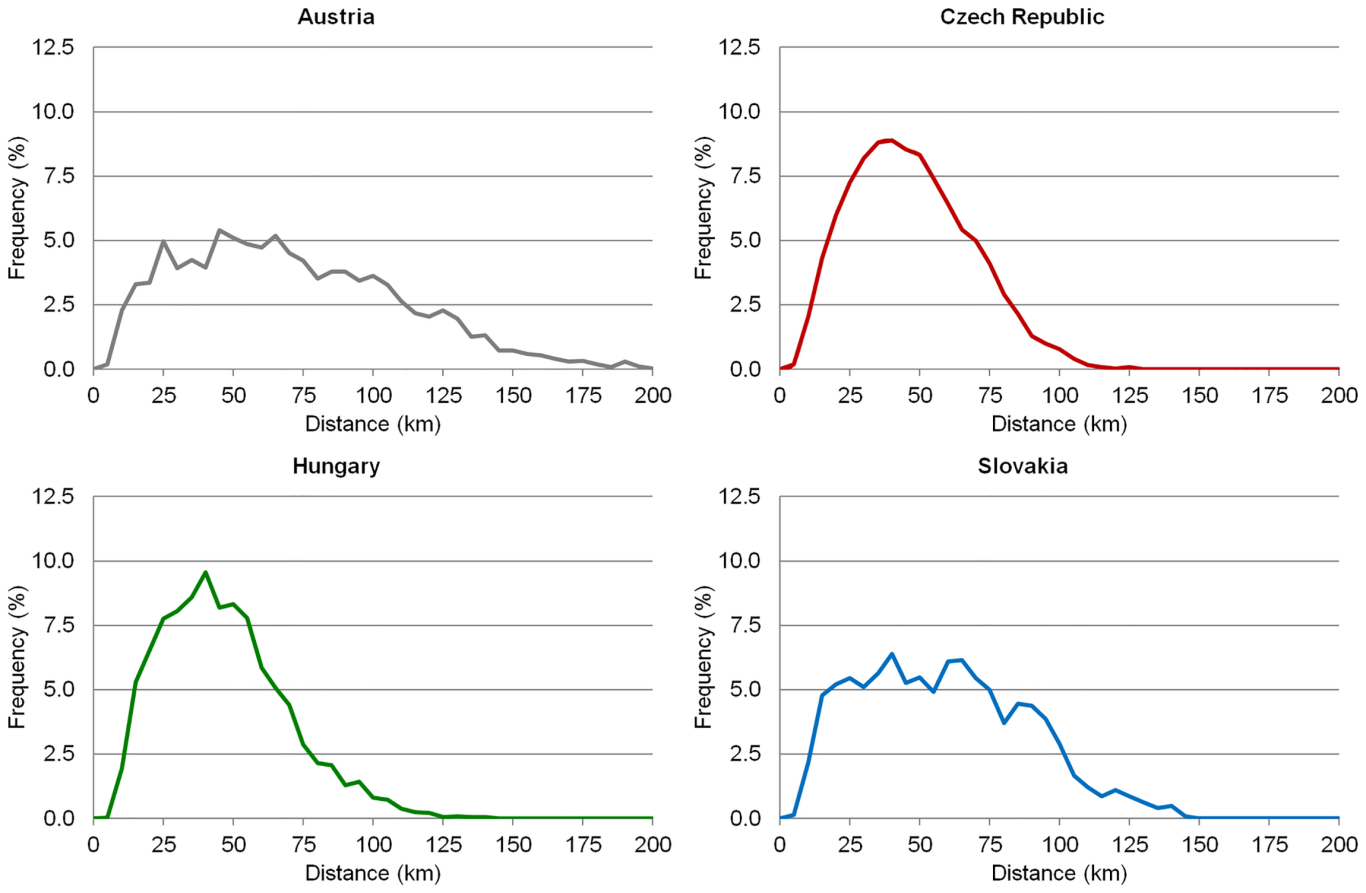
Distribution of municipalities according to the distance from NUTS 3 (NUTS 2 in Austria) centres. Source: own design.

Figs 7 and 8 show the time and metric accessibility from relevant municipalities to the centres of NUTS 3 regions in the Czech Republic, Hungary and Slovakia, and to the centres of NUTS 2 regions in Austria. In this case the differences between the four countries are the most distinct. The indirect role of physical geography (again terrain in particular) is significant in Austria and Slovakia. The influence of terrain and the consequent irregularities in settlement distribution is documented, particularly by the graph expressing the frequencies of municipalities according to the distance from regional administrative centres (Fig 8). Terrain is responsible for the most areas with poorer accessibility to regional capitals together with the eccentric locations of some—e.g. Graz (in Styria), Salzburg (in Salzburg) and Innsbruck (in Tyrol) in Austria, Banská Bystrica (in Banská Bystrica Region) in Slovakia. The eccentric location of regional capitals is also seen in individual cases in the Czech Republic (Olomouc in Olomouc Region) and Hungary (Kecskemét in Bács-Kiskun), although these two countries present very similar figures and patterns to each other but which differ from those recorded for Austria and Slovakia.

Austria is a well-documented example showing that terrain (relief) does not limit accessibility and spatial equity because of the level of its dissection. The accessibility of centres from their most distant areas or regions is favoured by a good transport infrastructure. However, there are distinct differences in the accessibility of centres when comparing individual Austrian regions particularly; a product of their different areas, which are also conditioned by historical developments. Most Austrian states (*bundesländer*) have their origins in the Middle Ages (apart from Vorarlberg—the 19^th^ century, Vienna and Burgenland—the 1920s). The borders of these units are also stable. They were and are primarily determined by physical geographical conditions, particularly the relief (mountain ranges). Therefore historical regions that resemble the current administrative regions were formed, which had an effect on, for instance, the distribution of slightly differentiated cultural population groups.

**Fig 9:**
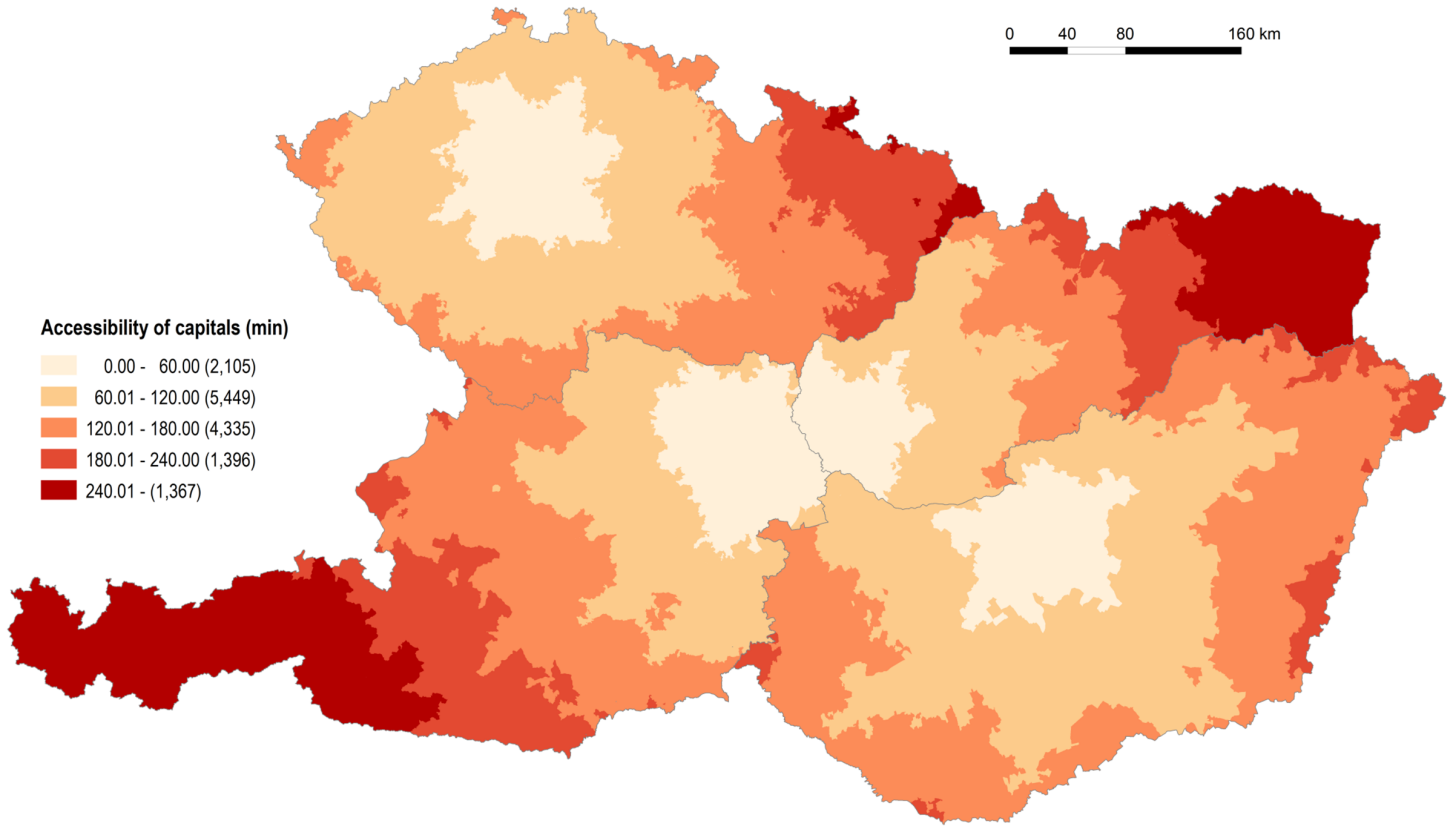
Accessibility of capital cities by individual road transport. Source: own design.

**Fig 10:**
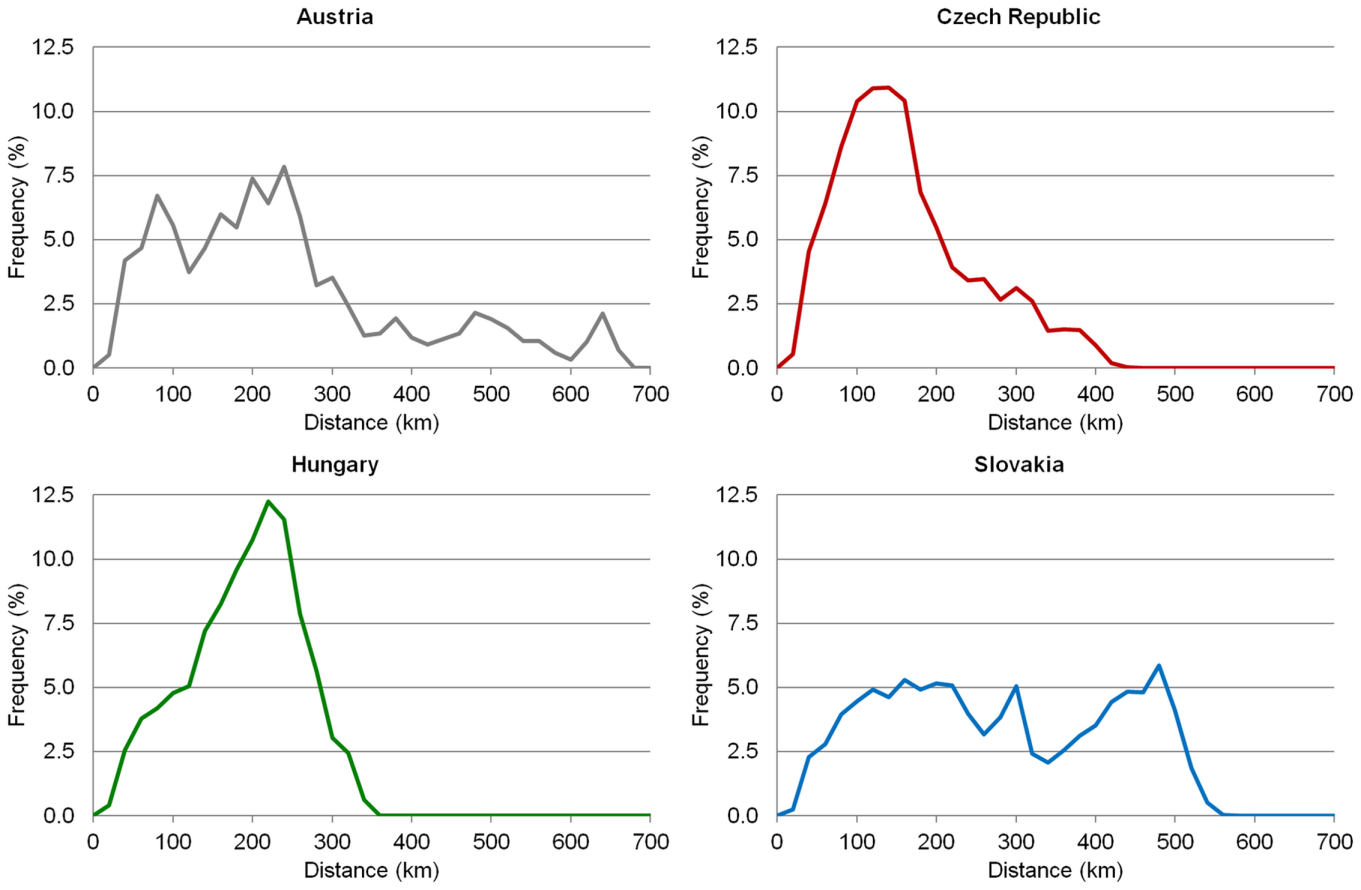
Distribution of municipalities according to the distance from capital cities. Source: own design.

Figs 9 and 10 show the time and metric accessibility of all municipalities to the country capitals (Vienna, Prague, Budapest, Bratislava). Again two cases are clearly visible and they are particularly caused by the location of capital cities, and secondarily by the shape of territory: the Czech Republic and Hungary on one hand, Austria and Slovakia on the other. Fig 10 shows that the accessibility of a capital city approximates to the most the ideal case presented in Fig 4 in the case of Hungary, whose capital is the most centrally located and whose shape is the most compact. Moreover the frictional effects of a space (distance) are the least important in the Hungarian territory thanks in particular to homogenous physical geographical features. All four countries have elongated shaped territories in an east-west direction. In this case the moderately eccentric location of the capital city in a north-south direction (Hungary) does not impair the spatial equity of its accessibility, the moderately eccentric location in an east-west direction (the Czech Republic) causes only moderate disadvantages for some areas, and a significantly eccentric location in an east-west direction (Austria, Slovakia) causes total asymmetry in the accessibility of the capital city (Fig 9).

**Fig 11:**
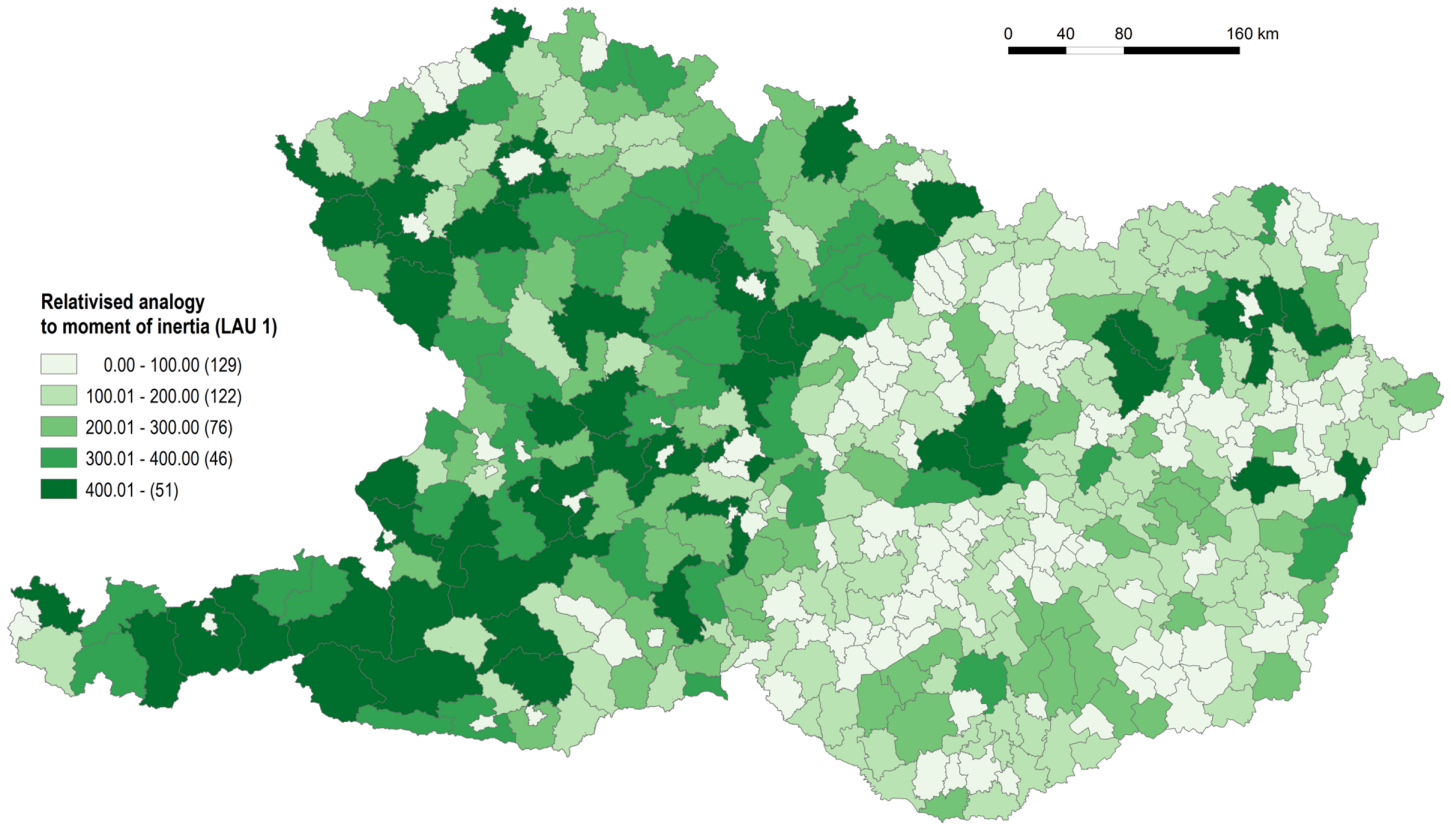
Measure of spatial equity for LAU 1 level. Source: own design.

**Fig 12:**
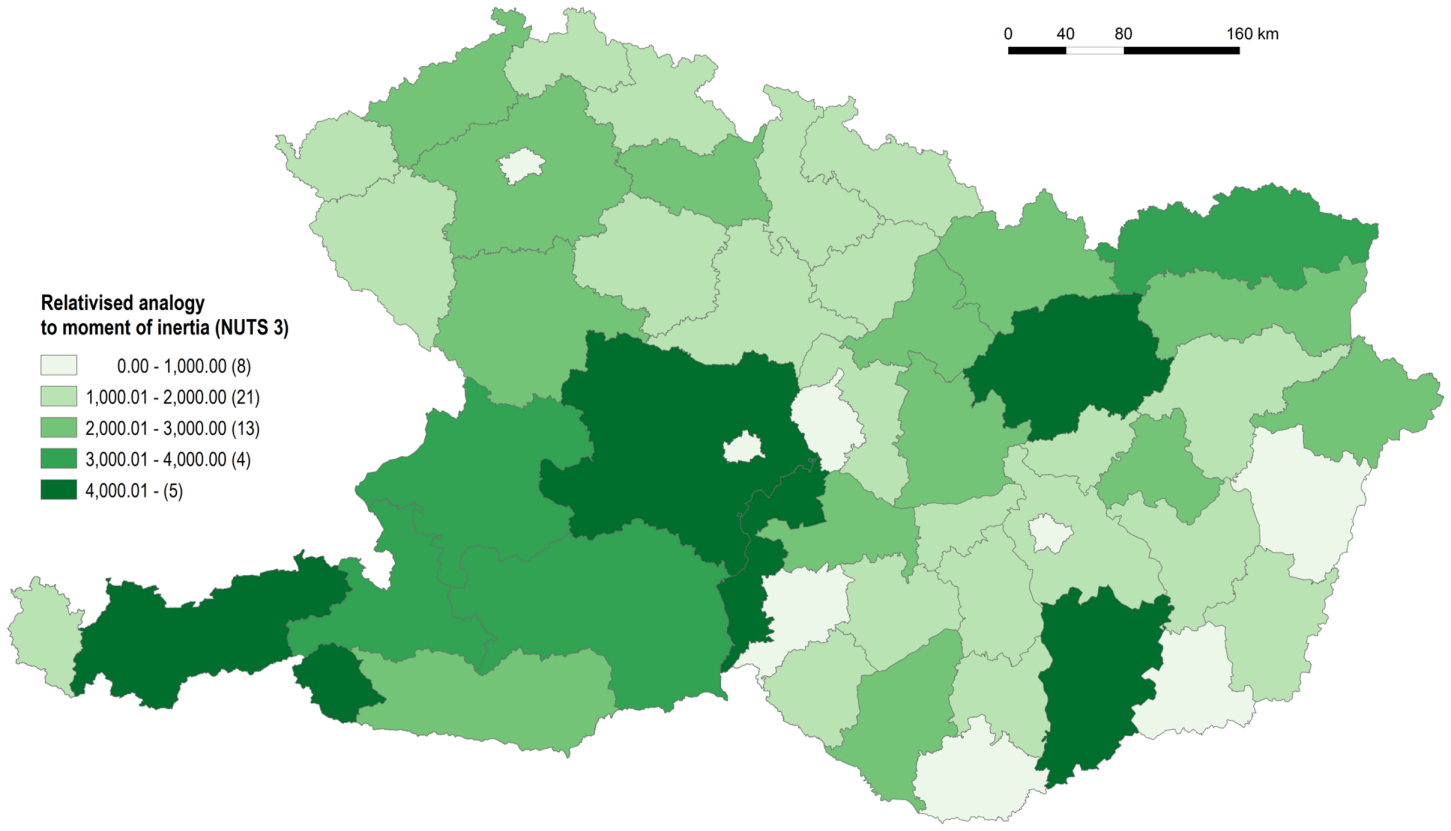
Measure of spatial equity for NUTS 3 (NUTS 2 in Austria) level. Source: own design.

As the preceding six figures have given the reader some basic insight into the issue of accessibility of administrative centres at three hierarchical levels, the following two maps, Figs 11 and 12 express the level of spatial equity using the relativised analogy to the moment of inertia *dmr* as a tool. Two significant facts can be inferred from the analysis of the LAU 1 level (Fig 11). The principle of spatial equity appears to be kept to less in Austria and the Czech Republic in comparison to Hungary and Slovakia. In the case of Austria it is particularly caused by the indirect role of physical geographical features that reduce especially the time accessibility of relevant centres and also by the greater size of less populated regions in the mountainous parts of territory. In the case of the Czech Republic it is caused by the size of the LAU 1 regions, and also by their shape in some cases.

The second interesting result appears in Slovakia along its border with Hungary. The reduced level of spatial equity in the LAU 1 regions concerned is a clear result of political malpractice dating back to the mid-1990s, which intentionally disadvantaged the Hungarian minority living in southern Slovakia. The administrative division of Slovakia is considered by many authors to be badly designed (see further). It was formed around the mid-1990s and is a product of the political situation and interests at the time of the Vladimír Mečiar government (1994–1998). Immediately after its introduction it was harshly criticised by the experts (e.g. [50, 51]). For example, the unsuitable and biased definition of LAU 1 (districts–*okresy*) does not respect the principles of functional affiliation and organisation of space. In the southern part of the territory the districts with the Hungarian population (Nové Zámky, Levice in Nitra Region; Rožňava, Trebišov in Košice Region etc.) are much larger than some of the districts in other parts of Slovakia (Bytča, Kysucké Nové Mesto, Turčianske Teplice in Žilina Region etc.). The principle of spatial equity is infringed considerably in this case [52].

As for the NUTS 3 (NUTS 2 in Austria) level, all three characteristics put forward in the theoretical section of the paper manifest their effect, as can be seen in Fig 12 (the causes are noted in brackets). A reduced level of spatial equity is seen in Tyrol (elongated shape, disjointed area), Lower Austria (size), and Burgenland (extremely elongated shape) in Austria; in Banská Bystrica Region (eccentric location of administrative centre, Banská Bystrica) in Slovakia; and finally in Bács-Kiskun (again eccentric location of administrative centre, Kecskemét) in Hungary.

## Conclusion

The paper has proposed a quantitative and rigorously “spatial” approach to the issue of spatial justice, or more precisely of spatial equity. The paper has assumed quite logically, but also grounded the assumptions in theory, that there are spatial (with some reserve “geometrical”) aspects of the equity concept that can be relatively easily quantified. Three such aspects have been suggested: the size of a region, the shape (compactness) of a region, and the location of the centre of the region. In order to prove their effects on the level of spatial equity, the concept of accessibility has been used and expressed both in metric and time distance, and also in the proportion of time and metric distance. The inclusion of time distance into the analysis has particularly important theoretical reasons and implications. The problem solved in the paper is not just reduced to a geometrical one, but the time distance also covers the character of a geographic space in terms of the homogeneity and heterogeneity of its physical geographical features, which are consequently mirrored for instance in the character and quality of transport networks. Thus the friction effects of a space on accessibility are handled in a more real context.

The theoretical assumptions put forward in the paper have been documented on the territory of four Central European countries (Austria, the Czech Republic, Hungary and Slovakia). The choice of these countries gives an opportunity to identify several frameworks for the analysis and interpretation. These countries present a variety of physical geographical conditions (Alpine Europe, Hercynian Europe, vast basin) and a political geographical and economic geographical development expressed by the existence of the Iron Curtain and by their development after its fall. In contrast the whole territory has a long tradition of being under common rule, and parts of the territory have an even longer common history (the split of Czechoslovakia dates back only to 1993, Hungary and Slovakia were united for almost a thousand years).

Even despite the great variety of contexts that can affect the interpretation of the results, the theoretical assumptions regarding the role of the size and shape of an administrative unit and the location of its centre have been corroborated, as well as the use of measures proposed to assess the level of spatial equity. Moreover, this corroboration has been based on the analysis at different hierarchical levels of administrative units (LAU 1, NUTS 3 or NUTS 2 in the case of Austria) and at the state level. The use of European nomenclature for administrative units has further supported the analysis in that it secured at least a basic level for inter-state comparisons, despite the different historical and political aspects of each country’s construction of its administrative division.

If spatial equity and spatial efficiency are considered as the two basic principles to be followed when delineating administrative regions, the paper looked more closely at the principle of spatial equity. However, the principle of spatial efficiency is equally important. In this case the delineation of administrative regions should be compared to a spatial distribution of economic activity. Some authors looked at this issue in the 1970s. For instance, [53] speaks of so-called functional urban regions, i.e. regions based on daily spatial population flows. Such regions are accurate images of the spatial organisation of an economy, and administrative regions should respect their delineation to a considerable extent. The issue of functional regions is also addressed in more recent works: from the point of view of their definition [54], their development in time [55] and the testing of their suitability for spatial analyses in comparison to administrative regions [37]. An analysis of the spatial efficiency of administrative regions using the existing functional spatial flows is well beyond the scope of the current paper and would need a further separate study.

## Supporting information

S1 Dataset SpatialEquity-Data. (XLSX)
